# Clinical Efficacy, Safety and Tolerability of a New Subcutaneous Immunoglobulin 16.5% (Octanorm [Cutaquig®]) in the Treatment of Patients With Primary Immunodeficiencies

**DOI:** 10.3389/fimmu.2019.00040

**Published:** 2019-02-04

**Authors:** Roger H. Kobayashi, Sudhir Gupta, Isaac Melamed, J. Fernando Mandujano, Ai Lan Kobayashi, Bruce Ritchie, Bob Geng, Thomas Prescott Atkinson, Syed Rehman, Eva Turpel-Kantor, Jiří Litzman

**Affiliations:** ^1^UCLA School of Medicine, Los Angeles, CA, United States; ^2^Division of Basic and Clinical Immunology, University of California, Irvine, Irvine, CA, United States; ^3^IMMUNOe Research Center, Centennial, CO, United States; ^4^Pediatric Pulmonary Associates of North Texas, Frisco, TX, United States; ^5^Midlands Pediatrics, Papillion, NE, United States; ^6^Division of Hematology, Department of Medicine, University of Alberta Hospital, Edmonton, AB, Canada; ^7^Divisions of Adult and Pediatric Allergy and Immunology, University of California, San Diego, La Jolla, CA, United States; ^8^Department of Pediatric Allergy, Asthma and Immunology, University of Alabama, Birmingham, AL, United States; ^9^Allergy and Asthma Center Inc., Toledo, OH, United States; ^10^Octapharma Pharmazeutika Produktionsges.m.b.H., Vienna, Austria; ^11^Department of Clinical Immunology and Allergology, St Anne's University Hospital in Brno, Faculty of Medicine, Masaryk University, Brno, Czechia

**Keywords:** primary immunodeficiencies, immunoglobulins, antibodies, SCIG, infections, infusion site reactions

## Abstract

**Introduction:** Subcutaneously administered immunoglobulin (SCIG) is increasingly used to treat patients with primary immunodeficiencies (PIDs). Octanorm (marketed as cutaquig® in USA and Canada) is a new 16.5% solution of human SCIG, manufactured by a process based on that of the intravenous preparation (IVIG) octagam®.

**Objectives:** To investigate the efficacy, safety and tolerability of octanorm in a prospective, open-label, single-arm phase 3 study involving adult and pediatric patients with PIDs (NCT01888484; clinicaltrials.gov/ct2/show/NCT01888484).

**Methods:** Patients who were previously treated with IVIG received a total of 64 weekly SCIG infusions, including 12 weekly infusions during the wash-in/wash-out period, followed by 52 weekly infusions during the evaluation period.

**Results:** A total of 61 patients aged 2–73 years received 3,497 infusions of octanorm. The mean dose per patient was 0.175 g/kg/infusion. The mean calculated dose conversion factor from the patients' previous IVIG dose for octanorm was 1.37. No serious bacterial infections developed during the study. The rate of other infections per person-year during the primary observation period was 3.43 (upper 95% CI 4.57). All but one non-bacterial infection were mild or moderate in intensity. IgG trough levels were constant during the course of the study. Eleven patients (18.0%) experienced 14 mild or moderate systemic adverse events (AEs) related to octanorm. The rate of related AEs per infusion was 0.004. In 76.7% of infusions, no infusion site reactions were observed and only two (0.3%) reactions were deemed severe. The incidence of site reactions decreased with successive infusions.

**Conclusion:** The new 16.5% SCIG octanorm was shown to be efficacious in preventing infections in PIDs, and was well tolerated.

## Introduction

Primary immunodeficiencies (PIDs) comprise a group of over 350 rare and potentially serious and life-threatening disorders (1). Most common and clinically significant PIDs are common variable immunodeficiency (CVID) and primary hypogammaglobulinemia, including X-linked agammaglobulinemia. Antibody deficiencies comprise more than 50% of PIDs (2). Individuals with antibody deficiencies suffer from recurrent bacterial and viral infections. If untreated, these can have severe complications, including organ failure and death (3, 4). Patients with antibody deficiencies require long-term immunoglobulin (IG) replacement therapy to prevent severe infections and their complications (3).

Both intravenous (IV) and subcutaneous (SC) IG administration (3) have been shown to be effective and safe, and have been in use for decades (5–9). SCIG infusions (10) have steadily replaced IVIG as a preferred method, particularly in children or where there are venous access issues or untoward systemic reactions from the IV route (11). SCIG can be self-administered or administered by the patients' caregiver. This allows greater freedom, convenience, as well as improved quality of life and treatment compliance (12).

Studies have demonstrated that the SC method of IG delivery is feasible, safe, efficient, and cost-effective (13–25). Patients receiving IVIG treatment can experience diminished beneficial effects toward the latter part of the infusion cycle as IgG levels decrease toward the nadir (trough level) (26–29). Pharmacokinetic (PK) studies together with IgG trough level determinations have demonstrated minimal fluctuation of serum IgG levels with SCIG treatment and higher trough serum levels than with IVIG treatment (22, 25, 30, 31). Another major benefit of SCIG is lower incidence of systemic adverse events (AEs) compared with IVIG (15, 24). With SCIG, local reactions at the infusion site are common, but typically are mild, decrease over time, and do not impede good tolerability of treatment (20).

Octanorm (marketed as cutaquig® in USA and Canada) (Octapharma AG, Lachen, Switzerland) is a new 16.5% solution of human immunoglobulin for SC administration, which utilizes an octagam®-based process (32). As with octagam®, pathogen removal steps include solvent/detergent treatment, incubation at pH 4.0, and validated pathogen removal during cold ethanol fractionation. The current study (NCT01888484) investigates efficacy, safety, tolerability, and pharmacokinetics of octanorm in adult and pediatric patients with PIDs. We report here efficacy, safety and tolerability of octanorm in the treatment of patients with PIDs.

## Materials and Methods

### Study Design

This prospective, open-label, single-arm phase 3 study was conducted at 18 centers in Europe and North America. Data collection on a small number of pediatric patients is ongoing.

Eligibility criteria included patients with PIDs [as defined by the European Society for Immunodeficiencies and the Pan-American Group for Immunodeficiency (33)] and patients with primary IgG deficiency (defined as depressed antibody levels below two standard deviations of the mean for age-matched standard controls) with clinically significant immunodeficiency. The patients were between the ages of 2 and 75 years and were receiving regular IVIG treatment with constant dosing for at least six prior infusions (with dosing between 0.20 and 0.80 g/kg body weight [BW] [±20% of the mean dose for the last six infusions]) with IgG trough levels ≥5.0 g/L for the last two IVIG infusions.

**Figure 1:**
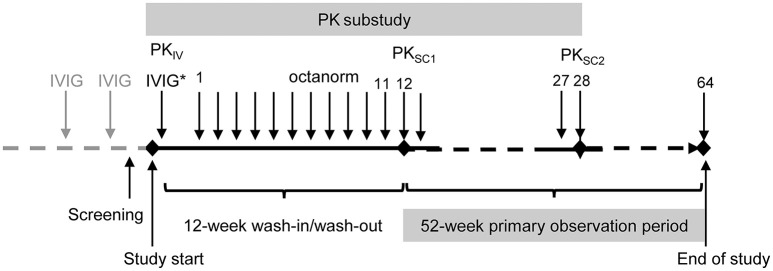
Study design. ^*^Study IVIG injection was only administered to the patients participating in the PK sub-study. PKIV, PK following IVIG prior to the switch to octanorm; PKSC1, PK after the 11th infusion of octanorm; PKSC2, PK after the 28th infusion of octanorm. IVIG, intravenous immunoglobulin; PK, pharmacokinetic; SCIG, subcutaneous immunoglobulin.

The study consisted of two phases (64 SCIG infusions in total): (A) a 12-weeks wash-in/wash-out period and (B) a 52-weeks primary observation period (Figure 1). To assess the bioavailability of total IgG after IVIG and SCIG administration, a PK sub-study was conducted. Early participants were enrolled in the PK sub-study and subsequently transitioned into the efficacy study; patients who enrolled later participated in the efficacy part of the study only. At enrollment, patients participating in the PK sub-study received a single dose of their previous IVIG to obtain a PK profile. IgG trough levels were measured at three different time points: after the last IVIG administration (PK_IV_), at the end of the 12 weeks wash-in/wash-out phase (PK_SC1_) and after the 28th SCIG administration (PK_SC2_).

All patients received weekly octanorm doses 1.5 times their previous monthly IVIG dose split into four equal weekly SCIG infusions, based upon published conversion rates for marketed SCIG products and the US Food and Drug Administration (FDA) requirements (16, 24, 34, 35). Patients/caregivers were taught to administer octanorm at home, with every 4th infusion given at the study site in order to confirm proper administration technique. Patients were required to maintain a diary documenting parameters related to infusion (date, volume, and speed of infusion), efficacy (occurrence of infections, missed days from work/school/day care, inpatient hospital stays), tolerability (local tissue reactions, body temperature 1 h after the end of the infusion), and health-related quality of life (HRQL; assessed using the Child Health Questionnaire-Parent Form [CHQ-PF50] for patients <14 years old and the SF-36 Health Survey for patients ≥14 years old). HRQL data were analyzed at weeks 1 and 28 and at the end of the study. AEs, concomitant medications and tolerability, including infusion site reactions, were recorded. Infections and infusion site reactions were classified as: mild (causes discomfort but does not interfere with the patient's routine activities), moderate (sufficiently discomforting to interfere with the patient's routine activities) or severe (incapacitating and prevents the pursuit of the patient's routine activities). IgG trough levels, analyzed at the local and the central laboratories, were collected from all patients during monitoring visits. A dose conversion factor (DCF) was calculated from the data obtained from 22 patients in the PK sub-study. DCF was calculated by dividing the area under the curve (AUC) of IVIG prior to the switch to octanorm by the AUC of SCIG after the 28th infusion of octanorm. Final assessments were performed 1 week after the completion of the last infusion or 1 week after premature withdrawal of the patient from the study. Systemic AEs [fever, myalgia, arthralgia] were recorded throughout the whole study. Patients were instructed to record all systemic AEs, as well as local infusion site reactions [swelling, tenderness, erythema] occurring up to 24 h after the end of each infusion, in the patient diaries. Any recorded events were transferred by investigators into the electronic system at each monitoring visit (every 4 weeks).

All efficacy and HRQL results are presented for the primary observation period. Octanorm exposure and AEs/tolerability are presented for the entire study. Efficacy and safety results were analyzed for the overall population and age groups: younger children (≥2 and <5 years), older children (≥5 and <12 years), adolescents (≥12 and <16 years) and adults (≥16 and ≤ 75 years). PK and HRQL analyses are presented for the combined population only, due to the limited number of children with these data available.

An Independent Data Monitoring Committee (IDMC) reviewed laboratory and clinical data sets with particular attention to thromboembolic events and clinically significant hemolysis at various intervals during the study.

This study was carried out in accordance with the recommendations of the FDA Guidance for Industry for IgG Studies and EMA guideline on the clinical investigation on SCIG and the international standards of Good Clinical Practice with written informed consent from all subjects. All subjects gave written informed consent in accordance with the Declaration of Helsinki. The protocol was approved by the National Competent Authorities, International Ethics Committees and Institutional Review Boards (IRBs, with two central IRBs used in the US).

### Statistical Analyses

All data were analyzed descriptively. Missing data were not imputed and calculations pertaining to person-year computations were based on collected values only. Serious bacterial infections (SBIs) included bacterial pneumonia, bacteremia/sepsis, osteomyelitis/septic arthritis, visceral abscess and bacterial meningitis. As no SBI was reported, no specific statistical analyses for SBIs were performed. The rate of other infections per person-year during the primary observation period is presented with the associated upper 95% confidence limit. Limiting the infection rates to the primary observation period ensures that infections can be attributed to steady-state treatment with octanorm unambiguously. The HRQL data are presented descriptively by visit, along with the change from baseline (first infusion). IgG plasma levels are summarized by infusion number over time. The DCF was determined from the observed AUC (after IVIG and SCIG administration) and the actual doses administered by means of linear least-square regression. To compare bioavailability of a patient's previous IVIG and octanorm, a two one-sided tests (TOST) analysis of the mean AUCτ ratio associated with the final adjusted SC versus the IV doses was performed. The TOST analysis for multiplicative equivalence of paired lognormal geometric means with bounds 0.8 and 1.25 was performed on the α = 0.05 confidence level.

## Results

### Study Population

**Figure 2:**
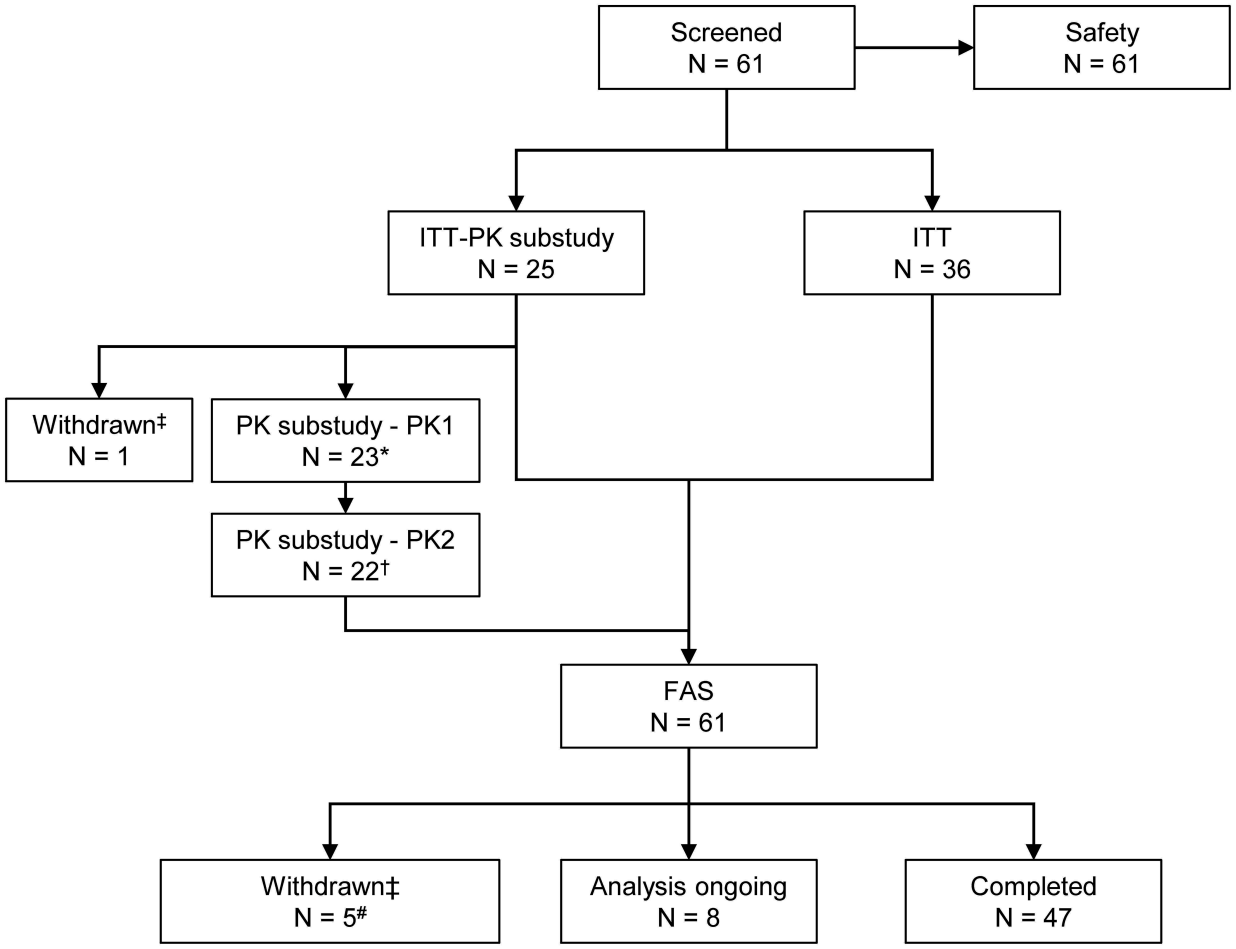
Patient disposition. ^*^One patient had no blood sampling and did not participate in the PK sub-study. ^†^One patient did not have PK samples available from the PK_SC2_ sampling time point. ^‡^The reason for withdrawal was the patient's decision in each case. ^#^Three patients withdrew before the start of the primary efficacy period. FAS, full analysis set; ITT, intention-to-treat; *N*, number of patients; PK, pharmacokinetic.

All 61 patients enrolled were included in the analysis, including four younger children (aged ≥2 to <5 years), 11 older children (aged ≥5 to <12 years), eight adolescents (aged ≥12 to <16 years) and 38 adults (aged ≥16 and <75 years). Six patients (three adolescents and three adults) withdrew voluntarily from the study for personal reasons; no withdrawal was due to AEs. A total of 47 patients (35 adult patients and 12 children and adolescents) completed the study (Figure 2, Supplementary Table 1), while in eight children and adolescents data collection was still ongoing (Supplementary Table 2).

The numbers of females and males were nearly equal, and the patients' ages ranged from 2 to 73 years. The majority of patients (86.9%) had a diagnosis of CVID (Table 1). Most patients' previous IVIG infusion schedule was every 4 weeks (47 patients; 77.0%). The mean dose of IVIG during the last 6 infusions prior to switching to SCIG was 0.44 g/kg every 3–4 weeks.

**Table 1:** Baseline characteristics.

	**Younger** **children** **≥2 years** **< 5 years** ***N* = 4**	**Older children** **≥5 years** **< 12 years** ***N* = 11**	**Adolescents** **≥12 years** **< 16 years** ***N* = 8**	**Adults** **≥16 years** **≤75 years** ***N* = 38**	**All patients** ***N* = 61**
Sex, %, (F/M)	25.0/75.0	18.2/81.8	37.5/62.5	71.1/28.9	54.1/45.9
Age, median (range), years	3.0 (2.0–4.0)	6.0 (5.0–10.0)	13.5 (12.0–15.0)	45.5 (16.0–73.0)	34.0 (2.0–73.0)
Body weight, median (range), kg	14.7 (13.0–23.4)	24.0 (19.0–56.0)	55.2 (48.4–86.4)	67.3 (44.3–98.6)	60.9 (13.0–98.6)
**ETIOLOGY OF PID**, ***N*** **(%)**					
CVID	1 (25.0)	7 (63.6)	8 (100.0)	37 (97.4)	53 (86.9)
XLA	1 (25.0)	2 (18.2)	0 (0.0)	0 (0.0)	3 (4.9)
Other^*^	2 (50.0)	2 (18.2)	0 (0.0)	1 (2.6)	5 (8.2)
**REGION**					
Europe	4 (100)	4 (36.4)	0 (0)	18 (47.4)	26 (42.6)
North America	0 (0)	7 (64.6)	8 (100)	20 (52.6)	35 (57.4)

* *“Other” includes: 1 case each of selective deficiency of IgG1 and IgG2 with deficiency of specific antibodies and hypogammaglobulinemia in younger children, 2 cases of hypogammaglobulinemia in older children and 1 case of IgG deficiency in an adult*.

*CVID, common variable immunodeficiency; F/M, female/male; N, number of patients; PID, primary immunodeficiency; XLA, X-linked agammaglobulinemia*.

### SCIG Administration Characteristics

Sixty-one patients received a total of 3,497 infusions of octanorm, with a median of 64 (mean 57.3) infusions per patient (range 2–65 infusions). The mean dose of octanorm administered per patient was 0.175 g/kg BW/infusion. The mean maximum volume per infusion site was 24.04 mL for adults and less for children and adolescents (10.25–16.01 mL/infusion site). The mean infusion flow rate was 22.86 mL/h/site and was likewise lower in children and adolescents (14.19–16.85 mL/h/site). As a general trend, the dose of octanorm per kg, duration of infusions, infusion volume, and in 1 infusion flow rate increased with age (Table 2).

**Table 2:** Octanorm dosing and infusion characteristics.

	**Younger** **children** **≥2 years** **< 5 years** ***N* = 4**	**Older children** **≥5 years** **< 12 years** ***N* = 11**	**Adolescents** **≥12 years** **< 16 years** ***N* = 8**	**Adults** **≥16 years** **≤75 years** ***N* = 38**	**All patients** ***N* = 61**
Number of infusions, *n*	240	658	285	2314	3497
Dose of octanorm administered, g/kg BW/patient	0.135 ± 0.055 (0.08–0.21)	0.160 ± 0.065 (0.06–0.29)	0.172 ± 0.047 (0.12–0.24)	0.185 ± 0.075 (0.09–0.39)	0.175 ± 0.069 (0.07–0.39)
Average duration of infusion,^*^ min	38.94 ± 6.64 (29.9-45.7)	65.73 ± 21.69 (36.1–106.3)	144.46 ± 59.80 (56.0–230.5)	111.46 ± 48.992 (45.2–304.5)	102.79 ± 52.73 (29.9–304.5)
**INFUSION VOLUMES AND FLOW RATES PER PATIENT**					
Maximum volume administered, mL	15.50 ± 6.61 (8–22)	31.36 ± 14.15 (9–62)	64.63 ± 17.37 (40–90)	77.74 ± 31.95 (34.5–159)	63.57 ± 34.23 (8–159)
Maximum volume administered/injection site, mL/site	10.25 ± 1.71 (8–12)	14.17 ± 4.21 (9–22.5)	16.01 ± 3.39 (13–22.5)	24.04 ± 6.47 (15–39.8)	20.30 ± 7.43 (8–39.8)
Maximum infusion flow rate, mL/h	25.00 ± 14.14 (15–45)	33.87 ± 10.41 (15–47)	38.14 ± 9.70 (28.2–50)	60.12 ± 17.34 (29.4–97.9)	50.20 ± 19.93 (15–97.9)
Maximum infusion flow rate/site (mL/h/site)	16.25 ± 4.33 (12.5–22.5)	16.85 ± 7.38 (3.8–30)	14.19 ± 6.12 (7.3–25)	22.86± 9.35 (10–51.8)	20.20 ± 8.98 (3.8–51.8)

*Data are mean ± SD, (range) except where indicated. N, number of patients; n, number of infusions; SD, standard deviation*.

* *Active infusion time only*.

Forty (65.9%) received at least 64 infusions and another 11.5% received 63 infusions. Fourteen patients (22.6%) received 62 or fewer infusions. One half of the patients (48.6 %) used one infusion site only, while a quarter each used two (24.4%) or three (27.0%) sites. The majority of patients (46/61; 75.4%) did not have more than two infusions outside of the treatment window of ± 2 days, with 38 (62.3%) patients administering all infusions within the treatment window.

### Efficacy

No SBIs occurred in any patients during the entire study period (wash-in/wash-out and primary observation periods). A total of 188 other (viral and non-serious bacterial) infections were observed in 52 (85.2%) patients in the primary observation period (Table 3). The rate of other infections per person-year was 3.432 (upper 95% CI 4.572). The most frequently observed were upper respiratory tract infections (108 infections), infections not elsewhere classified, such as influenza and sore throat (20 infections), and infections of the lower respiratory and genitourinary tract (19 infections each). Infections reported in the children were principally ear infections, infection of the upper and lower respiratory tract, gastrointestinal tract, and of the genitourinary tract. In adults, upper respiratory tract, gastrointestinal tract and the genitourinary tract infections were most frequently reported.

**Table 3:** Efficacy parameters during treatment with octanorm.

	**Younger** **children** **≥2 years** **< 5 years** ***N* = 4**	**Older children** **≥5 years** **< 12 years** ***N* = 11**	**Adolescents** **≥12 years** **< 16 years** ***N* = 8**	**Adults** **≥16 years** **≤75 years** ***N* = 38**	**All patients** ***N* = 61**
Any infection, *N* (%) n^*^	4 (100.0) 13	9 (81.8) 40	5 (62.5) 11	34 (89.5) 124	52 (85.2) 188
Mild infections	4 (100.0) 13	9 (81.8) 37	5 (62.5) 6	30 (78.9) 80	48 (78.7) 136
Moderate infections	0 (0.0) 0	3 (27.3) 3	3 (37.5) 4	20 (52.6) 44	26 (42.6) 51
Severe infections	0 (0.0) 0	0 (0.0) 0	1 (12.5) 1	0 (0.0) 0	1 (1.6) 1
Rate of infections per person-year	3.47	3.92	2.58	3.39	3.43
One-sided 95% CI—upper limit	8.41	7.20	5.94	4.91	4.57
Fever episodes, *n* (rate per person-year)	0	2 (0.20)	1 (0.24)	3 (0.08)	6 (0.11)
Absences from school/work, days (rate per person-year)	NA	47 (4.61)	15 (3.52)	72 (1.20)	134 (2.63^†^)
Hospitalizations due to infection, days (rate per person-year)	0	0	2 (0.47)	0	2 (0.04)

*During primary observation period*.

* *Mild: causes discomfort but does not interfere with the patient's routine activities; moderate: sufficiently discomforting to interfere with the patient's routine activities; severe: incapacitating and prevents the pursuit of the patient's routine activities*.

† *Based on 57 patients; four younger children were not included in this analysis as it was not applicable to them*.

*CI, confidence interval; N, number of patients; n, number of infections; NA, not applicable*.

Over three quarters of the infections were mild (78.7%) and one quarter (27.1%) were moderate in intensity (see Table 3 for severity definitions). An adolescent patient required hospitalization for bronchiolitis due to non-bacterial severe respiratory syncytial virus (RSV) infection.

The rate of febrile episodes per person-year was 0.11, and it did not vary among all age groups. The total number of days missed from school/work was 134, resulting in a rate of 2.63 days per patient year. One adolescent patient was hospitalized for RSV infection, resulting in a total of 0.04 days of hospitalization for infection per person year.

During the efficacy evaluation period, antibiotics, including systemic and topical, were administered to 41 (67.2%) patients (Table 4), with an average of 2.1 treatment episodes per patient-year. Eight patients received prophylactic antibiotic treatment for indications such as acne, short bowel syndrome, chronic lung disease or chronic respiratory problems. The annualized duration of antibiotic treatment was 51.77 days. The annualized duration of systemic antibiotic treatment was 39.62 days.

**Table 4:** Systemic and topical antibiotic use, overall and by region.

	**Younger** **children** **≥2 years** **< 5 years**	**Older children** **≥5 years** **< 12 years**	**Adolescents** **≥12 years** **< 16 years**	**Adults** **≥16 years** **≤75 years**	**All patients**
**SYSTEMIC AND TOPICAL ANTIBIOTIC USE**					
Overall, N	4	11	8	38	61
Patients with antibiotic treatment, *N* (%)	3 (75.0)	7 (63.6)	4 (50.0)	27 (71.1)	41 (67.2)
Rate of treatment episodes per person-year	3.20	1.57	1.41	2.27	2.14
Rate of treatment days per person-year	29.62	50.29	96.00	49.28	51.77
North America, *N*	0	7	8	20	35
Patients with antibiotic treatment, *N* (%)	0	3 (42.9)	4 (50.0)	18 (90.0)	25 (71.4)
Rate of treatment episodes per person-year	0	0.95	1.41	3.07	2.36
Rate of treatment days per person-year	0	53.40	96.01	68.57	69.29
Europe, *N*	4	4	0	18	26
Patients with antibiotic treatment, *N* (%)	3 (75.0)	4 (100.0)	0	9 (50.0)	16 (61.5)
Rate of treatment episodes per person-year	3.20	2.58	0	1.48	1.89
Rate of treatment days per person-year	29.62	45.20	0	30.01	32.23
**SYSTEMIC ANTIBIOTIC USE**					
Overall, *N*	4	11	8	38	61
Patients with antibiotic treatment, *N* (%)	3 (75.0)	7 (63.6)	4 (50.0)	26 (68.4)	40 (65.6)
Rate of treatment episodes per person-year	3.20	1.27	1.41	2.13	1.99
Rate of treatment days per person-year	29.61	48.72	96.01	31.53	39.62
North America, *N*	0	7	8	20	35
Patients with antibiotic treatment, *N* (%)	0	3 (42.9)	4 (50.0)	18 (90.0)	25 (71.4)
Rate of treatment episodes per person-year)	0	0.95	1.41	2.96	2.29
Rate of treatment days per person-year	0	53.40	96.01	48.82	56.79
Europe, *N*	4	4	0	18	26
Patients with antibiotic treatment, *N* (%)	3 (75.0)	4 (100.0)	0	8 (44.4)	15 (57.7)
Rate of treatment episodes per person-year	3.20	1.81	0	1.31	1.66
Rate of treatment days per person-year	29.62	41.07	0	14.27	20.49

*N, number of patients*.

When summarized by region, adult patients in North America were more likely to be treated with antibiotics (90.0% of patients in the US vs. 50.0% in Europe), on more occasions (3.1 vs. 1.5 treatment episodes per person-year) and for longer time (68.6 vs. 30.0 treatment days per person-year) than adult patients in Europe. The number of children and adolescents were too few to derive statistically valid data.

### IgG Plasma Levels

**Figure 3:**
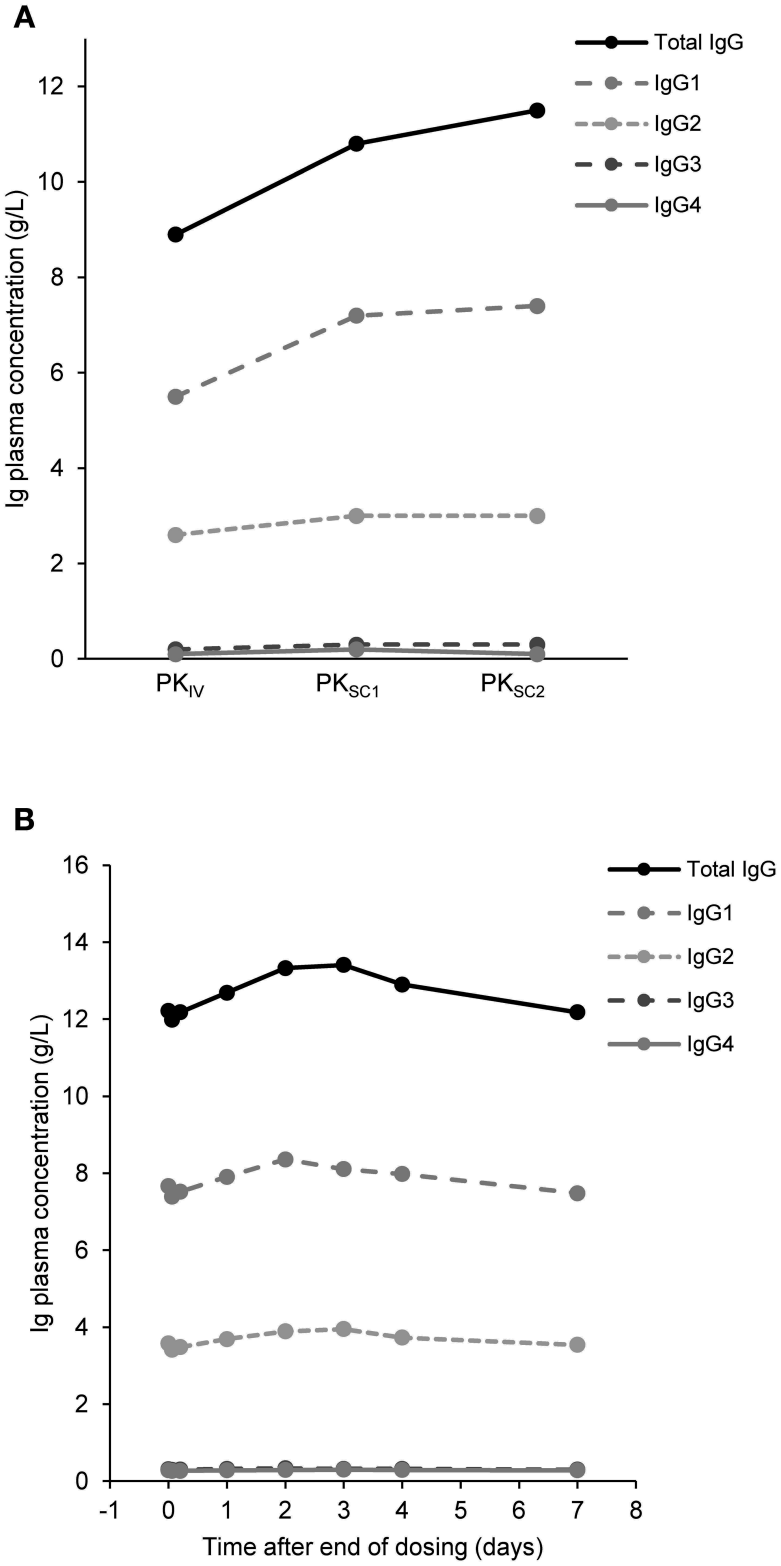
IgG concentrations over time. **(A)** Median trough concentrations of IgG and its subclasses after IVIG infusion (PK_IV_) and after octanorm infusions at 11 (PK_SC1_) and 28 weeks (PK_SC2_). For PK_IV_ and PK_SC1_ *N* = 22, for PK_SC2_ *N* = 21. N, number of patients; IgG, immunoglobulin G; PK_IV_, PK following IVIG prior to the switch to octanorm; PK_SC1_, PK after the 11th infusion of octanorm; PK_SC2_, PK after the 28th infusion of octanorm—at steady state. **(B)** IgG levels over 7 days after infusion. Data are shown for the 22 patients who participated in the PK sub-study and had IgG measurements at week 28 (PK_SC2_).

Twenty-two patients who participated in the PK sub-study had IgG trough levels determined at three different time points. Nineteen patients were adults, one was aged between 12 and 16 years and two were aged between 6 and 12 years (Supplementary Table 1). Total IgG and IgG subclass trough levels did not fluctuate during the course of the study, with higher trough levels achieved with octanorm treatment compared with their previous IVIG (Figure 3A). There were no patients with IgG trough levels below 5 g/L for both IVIG and SCIG. Analysis of plasma concentrations of IgG and IgG subclasses after infusion of octanorm at 28 weeks showed notably flat plasma PK profiles (Figure 3B).

### IVIG and SCIG Dosing and IgG Bioavailability

The DCF used in the study was 1.5 times the patients' previous IVIG dose divided over 4 weeks. Based on the actual dosing of IVIG and SCIG in the patients with PK determinations (*N* = 22), the mean calculated ratio for DCF was 1.370, and the geometric mean was 1.232.

The geometric mean of SCIG AUC at PK_SC2_/IVIG AUC was 1.0253, (90% CIs: 0.9778, 1.0751), thus confirming similar bioavailability between IVIG and SCIG when the DCF of 1.5 was applied.

### Patient Experience

There were no major changes in the average HRQL CHQ-PF50 scores (in pediatric patients younger than 14 years) over time but the number of patients (parents) completing the questionnaire was low (10/19 responded at the end-of-study visit). For patients aged 14 years and older the HRQL SF-36 questionnaire was used. The mean scores increased throughout the study for both summary scores (physical health and mental health) and for 7 of the 8 scales (there was a mean decrease of 0.03 for bodily pain). The largest increase was seen in the general health score (mean increase 3.97 points).

### Safety and Tolerability

Eleven patients (18.0%) had a total of 14 systemic AEs (excluding local infusion site reactions) that were considered to be related to octanorm; none were classed as serious (Table 5). The rate of related AE per infusion was 0.0040. Three transient, related AEs (myalgia, abdominal swelling, and transient positive direct Coombs test) were judged as moderate and the remaining were mild. No related AEs were reported in the younger children. None of the related AEs resulted in a change of octanorm dose or in withdrawal from the study. One patient had a transiently positive Coombs test, which resolved. Further, no reported evidence of hemolysis occurred, with no patient experiencing a concurrent positive Coombs test and a drop in hemoglobin. There was no indication of thrombosis during the study. Two patients had free hemoglobin present and one patient had increased hemoglobin and decreased haptoglobin levels. However, all four changes were considered mild and transient. Evaluation of vital signs and laboratory tests did not raise any safety concerns. There were no AEs leading to death or withdrawal from the study.

**Table 5:** Related adverse events (excluding infections and infusion site reactions).

	**Younger** **children** **≥2 years** **< 5 years** ***N* = 4** ***N* (%) *n***	**Older children** **≥5 years** **< 12 years** ***N* = 11** ***N* (%) *n***	**Adolescents** **≥12 years** **< 16 years** ***N* = 8** ***N* (%) *n***	**Adults** **≥16 years** **≤75 years** ***N* = 38** ***N* (%) *n***	**All patients** ***N* = 61** ***N* (%) *n***
Any Related AE	–	2 (18.2%) 3	1 (12.5%) 1	8 (21.1%) 10	11 (18.0%) 14
Headache	–	1 (9.1%) 1	–	1 (2.6%) 2	2 (3.3%) 3
Abdominal distension	–	–	–	1 (2.6%) 1	1 (1.6%) 1
Abdominal pain upper	–	–	–	1 (2.6%) 1	1 (1.6%) 1
Vomiting	–	1 (9.1%) 1	–	–	1 (1.6%) 1
Myalgia	–	–	1 (12.5%) 1	–	1 (1.6%) 1
Pyrexia	–	–		1 (2.6%) 1	1 (1.6%) 1
Body temperature increased	–	1 (9.1%) 1	–	–	1 (1.6%) 1
Coombs direct test positive^*^	–	–	–	1 (2.6%) 1	1 (1.6%) 1
Free hemoglobin present	–	–	–	2 (5.3%) 2	2 (3.3%) 2
Hemoglobin increased	–	–	–	1 (2.6%) 1	1 (1.6%) 1
Haptoglobin decreased	–	–	–	1 (2.6%) 1	1 (1.6%) 1

* *The patient with a positive Coombs direct test did not have hemoglobin decrease of ≥2 g/dL*.

*AE, adverse event; N, number of patients with AEs; n, number of events*.

### Infusion Site Reactions

No localized site reactions were observed for three-quarters (76.7%; 2683/3497) of analyzed infusions. A mild reaction was observed in one-fifth of the infusions (20.8%; 728/3497); a moderate reaction in 2.4% (84/3497) of infusions, and a severe reaction was observed for only two infusions (0.1%). The most common site reactions were erythema (77.1% of patients), swelling (37.7%), and pruritus (23.0%). The majority of the reactions were mild (89.4%) or moderate (10.3%) in intensity. Of the two severe reactions (0.3%), one was bruising at the injection site and the other a self-reported allergic reaction at a lower back injection site, which was assessed as severe by the patient during home administration but could not be confirmed as severe by the site investigator or other medical personnel during the visit at the study center a few days later. Neither patient experienced any systemic adverse reactions to the infusions.

**Figure 4:**
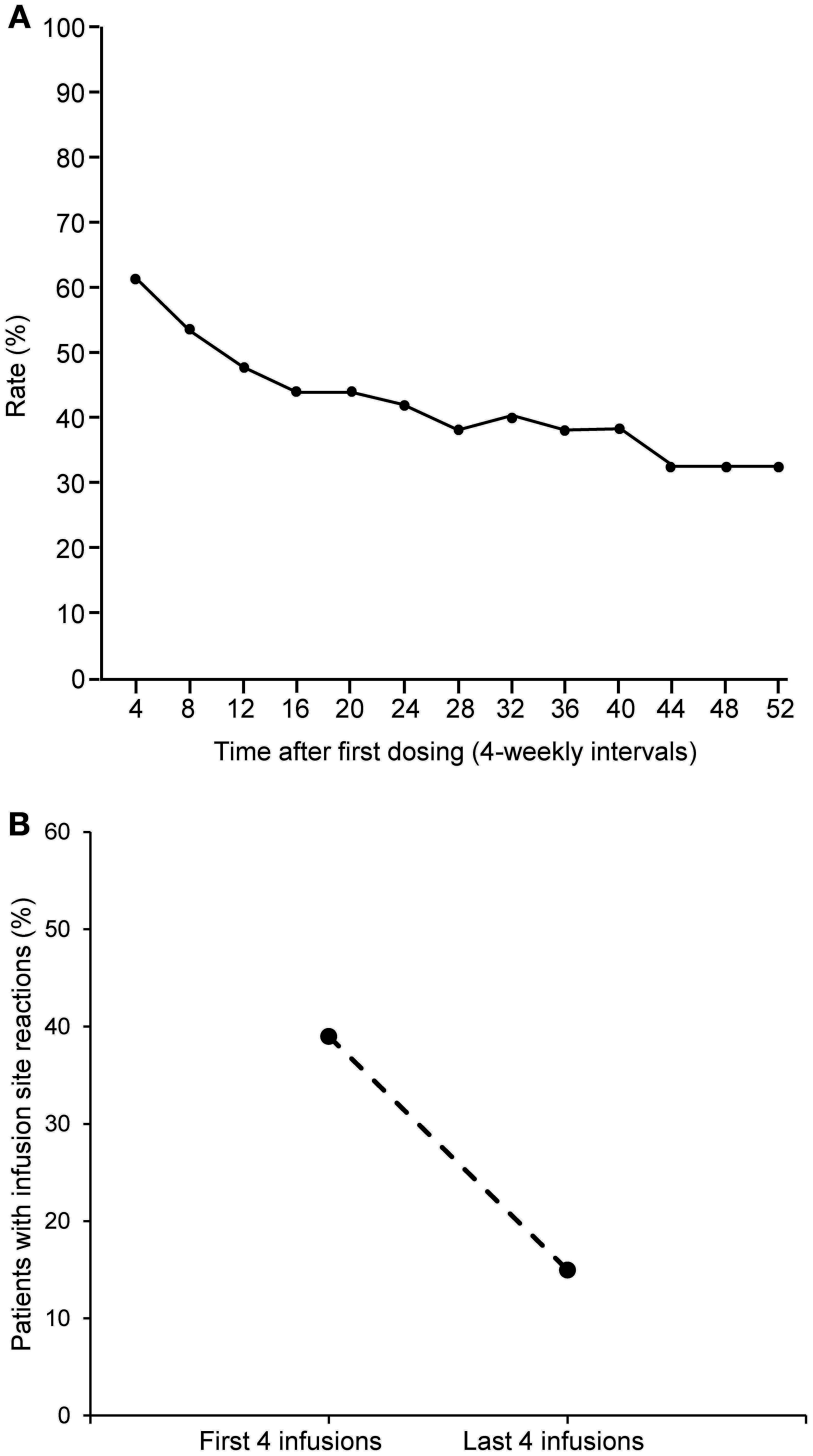
Rate of infusion site reactions over time. **(A)** Rate of infusion site reactions over time. **(B)** Comparison of the rate of infusion site reactions in the first and last 4 weeks of the study.

**Figure 5:**
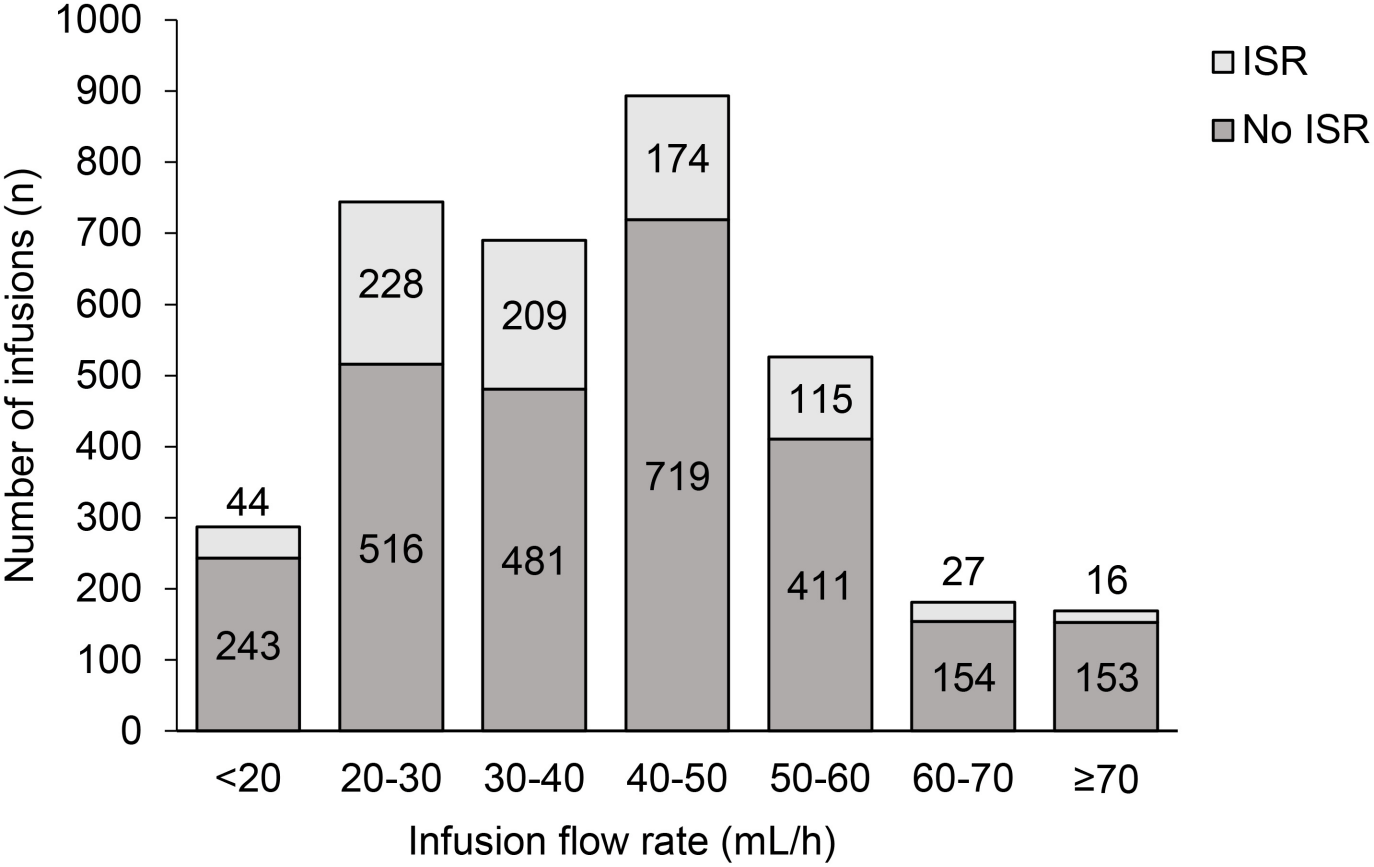
Infusion flow rate and infusion site reactions. ISR, infusion site reaction.

The percentage of infusions associated with site reactions decreased over time (Figure 4A), and the proportion of patients experiencing site reaction decreased from 39% in the first 4 weeks down to 15% in the last 4 weeks (Figure 4B). When two or more infusion sites were used, the total volume and rate of infusion shown are the sum of all the infusion sites used. This total volume and rate did not result in increased systemic reaction (Figure 5). The highest proportion of reactions was observed at infusion rates of 20–30 mL/h (228/744; 44.1%) and 30–40 mL/h (209/690; 30.2%) and the lowest with 70 mL/h or faster (16/169; 9.5%) and 60–70 mL/h (27/181; 14.9%).

## Discussion

Octanorm is a new SCIG manufactured by a process based on IVIG octagam®, including identical virus elimination techniques (36). In this study, it was demonstrated that patients on a stable dose of IVIG can be safely transitioned to SCIG octanorm, with SCIG treatment providing very good protection from infection and steady IgG plasma levels.

Patients in this study were on a stable dose of IVIG prior to the transition to SCIG. Individual doses for SCIG were predicated on the patients' prior stabilized IVIG dose and calibrated by a factor of 1.5, which was standard at the time of the protocol creation, to compensate for differences in bioavailability between the two infusion methods (16, 24, 34). The actual DCF calculated from the plasma IG levels after IV and SC infusion was 1.370, close to the presumptive conversion factor and in line with the current dosing recommendations for other licensed 16 and 20% SCIG products (24, 37, 38). When the AUCs after IVIG and SCIG at steady state (week 28) were compared, the ratio was 1.0253, confirming bioequivalence of the two treatment approaches when the DCF of 1.5 was applied.

The mean (SD) dose per infusion of octanorm in this study was 0.175 (0.069) g/kg BW and ranged from 0.07 to 0.39 g/kg BW. Maximum infusion volume and infusion flow rate were lowest in the younger children (15.50 mL/h and 25.00 mL) and highest in adults (60.12 mL/h and 77.74 mL), as would be expected based on the differences in body weight. Doses reported for a previously licensed 20% SCIG product, IgPro20, ranged from 0.179 to 0.224 g/kg BW (16) and the reported mean (SD) dose for Immune Globulin Subcutaneous (Human), 20% Solution was 0.222 (0.071) g/kg BW (39). The mean dose of another 16% SCIG product was reported as 0.0898 g/kg BW (20). Doses administered in the current study led to high trough levels of plasma IgG over the entire study period (a median of 11.5 g/L before the infusion at week 28). Additionally, the lower viscosity of octanorm compared with 20% IG products has been shown to improve injectability (ease of injecting the solution by a syringe) of the product, requiring less force and easier handling for administration (manuscript in preparation) (40, 41). This may be of particular benefit for elderly patients and patients suffering from hand dexterity problems.

In line with the high steady-state IG plasma levels, octanorm was highly effective at preventing infections, with no SBIs observed and a rate of 3.432 per patient/year for other infections. Previously published infection rates show a yearly rate per patient of 5.18 (95% CI 4.305, 6.171) for IGSC 20% (39), 2.76 for IgPro20 (16), 3.95 for a 16% SCIG (20) and 4.1 for a 10% SCIG (25). Although it is difficult to compare efficacy results among studies due to differences in patient populations and study designs, infection rates observed in the study with octanorm are in line with those reported for other SCIGs. Additional data gathered in the study, including episodes of fever, hospitalizations, absences from school/work, and HRQL questionnaire responses, further confirmed the efficacy of octanorm.

In general, there were no notable differences between children and adults in any of the efficacy parameters examined, showing that octanorm is appropriate for use in PID patients regardless of their age.

IG replacement therapy has been demonstrated to markedly reduce the number of severe infections and diminish the use of frequent antibiotics; however, IG cannot completely eliminate the use of antibiotics, even in those patients on maintenance IG therapy. In this study, we observed higher concomitant use of antibiotics in North American than in European centers (90.0 vs. 50.0% of adult patients). For systemic and topical antibiotics, we show 69.29 treatment days/patient-year in the US and 32.23 in Europe. If only systemic antibiotics use is analyzed, this decreased to 56.79 days/ patient-year in the US and 20.49 days per person-year in the European centers, indicating that in a low number of adult patients non-systemic antibiotics were administered for relatively long periods. This is in contrast to what has been previously observed in a study with a 20% SCIG, which noted use of concomitant antibiotics for 48.5 days/patient-year in the US centers and for 72.75 days/patient-year in European centers (40, 42). In surveys of members of the European Society for Immunodeficiency (ESID) and the American Academy of Allergy, Asthma and Immunology (AAAAI) it also appeared that the use of adjunct antibiotic treatment is more prevalent in European centers (43).

One of the major advantages of SCIG over IVIG is that subcutaneous delivery of IG is associated with fewer systemic AEs. On the other hand, local reactions at SC injection sites are common. These reactions are rarely severe, and are tolerated by most patients. In the meta-analysis by Orange et al. the reporting rate varied from 0.028 to 0.697 per infusion demonstrating that the majority of patients tolerate SCIG well (34). In the current study, 23% of infusions were associated with an infusion site reaction. Reactions were almost exclusively mild or moderate and their incidence decreased over time.

The rate of systemic AEs attributed to octanorm was low (0.23 related AEs per patient). While it is difficult to compare safety and tolerability of different SCIG preparations from different studies due to differences in study design, dosing and reported parameters (44), the low incidence of related AEs and infusion site reactions in this study points to excellent safety and tolerability of octanorm.

One limitation of this study is the use of concomitant antibiotics, which may have an effect on infection frequency. However, this complex issue has not been addressed in any controlled studies. Additionally, as this was a multi-center study, clinical practice might have differed at different sites and across geographical regions. There may have been some variation in the patients' condition.

In conclusion, the new 16.5% SCIG octanorm had a favorable safety profile and was well tolerated by pediatric and adult PID patients. The majority of infusion site reactions were mild and decreased in frequency over time. Octanorm also demonstrated excellent protection from infection, with no SBIs and a low rate of other infections.

## Disclosure

RK reports grants, personal fees, and non-financial support from Octapharma AG during the conduct of the study; grants and personal fees from Baxalta/Shire, grants from Vietnam Respiratory Society, Hanoi Vietnam, grants from Vietnam National Children's Hospital Hanoi, Vietnam, personal fees from UCLA School of Medicine outside the submitted work. SG reports grants from Octapharma AG during the conduct of the study; grants from Octapharma AG, Shire, CSL Behring, ProMatic, and BPL (US) outside the submitted work. IM reports grants and other funding from Octapharma AG during the conduct of the study, and grants and other funding from Octapharma AG outside the submitted work. JM reports personal fees from Octapharma AG outside of the submitted work. AK reports grants from Octapharma AG during the conduct of the study and grants from Octapharma AG outside the submitted work. BR reports funding for Investigator Initiated studies (University of Alberta Biobank) from Baxter, CSL Behring, Novartis, Novo Nordisk, Pfizer, Wyeth outside of the submitted work. BG reports grants from Octapharma AG during the conduct of the study; personal fees from CSL Behring, Shire, RMS, and Horizon and grants from Grifols and Shire outside of the submitted work. TA reports other funding from Baxalta/Shire outside of the submitted work. ET-K is an employee of Octapharma Pharmazeutika Produktionsges.m.b.H. JL reports personal fees from Octapharma Pharmazeutika Produktionsges.m.b.H. during the conduct of the study and personal fees from Shire Plc outside the submitted work.

## Author Contributions

All authors listed have made a substantial, direct and intellectual contribution to the work, and approved it for publication.

### Conflict of Interest Statement

The authors declare that the research was conducted in the absence of any commercial or financial relationships that could be construed as a potential conflict of interest.
