# Corrigendum: Clinical efficacy, safety and tolerability of a new subcutaneous immunoglobulin 16.5% (Octanorm [Cutaquig^®^]) in the treatment of patients with primary immunodeficiencies

**DOI:** 10.3389/fimmu.2022.1110388

**Published:** 2022-12-20

**Authors:** Roger H. Kobayashi, Sudhir Gupta, Isaac Melamed, J. Fernando Mandujano, Ai Lan Kobayashi, Bruce Ritchie, Bob Geng, Thomas Prescott Atkinson, Syed Rehman, Eva Turpel-Kantor, Jiří Litzman

**Affiliations:** ^1^UCLA School of Medicine, Los Angeles, CA, United States; ^2^Division of Basic and Clinical Immunology, University of California, Irvine, Irvine, CA, United States; ^3^IMMUNOe Research Center, Centennial, CO, United States; ^4^Pediatric Pulmonary Associates of North Texas, Frisco, TX, United States; ^5^Midlands Pediatrics, Papillion, NE, United States; ^6^Divisionof Hematology, Department of Medicine, University of Alberta Hospital, Edmonton, AB, Canada; ^7^Divisions of Adult and Pediatric Allergy and Immunology, University of California, San Diego, La Jolla, CA, United States; ^8^Department of Pediatric Allergy, Asthma and Immunology, University of Alabama, Birmingham, AL, United States; ^9^Allergy and Asthma Center Inc., Toledo, OH, United States; ^10^Octapharma Pharmazeutika Produktionsges.m.b.H., Vienna, Austria; ^11^Department of Clinical Immunology and Allergology, St Anne’s University Hospital in Brno, Faculty of Medicine, Masaryk University, Brno, Czechia

**Keywords:** primary immunodeficiencies, immunoglobulins, antibodies, SCIG, infections, infusion site reactions

In the published article, there was an error in **Table 4** as published. The table shows the rate of treatment days per person-year in adults (≥16 years and ≤75 years) as 148.82. The correct number is 48.82. The corrected **Table 4** and its caption “Systemic and topical antibiotic use, overall and by region” appear below.

**Table 4:** Systemic and topical antibiotic use, overall and by region.

	Younger children ≥2 Years<5 Years	Older children ≥5 Years<12 Years	Adolescents ≥12 Years<16 Years	Adults ≥16 Years≤75 Years	All patients
Systemic and topical antibiotic use					
Overall, N	4	11	8	38	61
Patients with antibiotic treatment, N (%)	3 (75.0)	7 (63.6)	4 (50.0)	27 (71.1)	41 (67.2)
Rate of treatment episodes per person-year	3.20	1.57	1.41	2.27	2.14
Rate of treatment days per person-year	29.62	50.29	96.00	49.28	51.77
North America, N	0	7	8	20	35
Patients with antibiotic treatment, N (%)	0	3 (42.9)	4 (50.0)	18 (90.0)	25 (71.4)
Rate of treatment episodes per person-year	0	0.95	1.41	3.07	2.36
Rate of treatment days per person-year	0	53.40	96.01	68.57	69.29
Europe, N	4	4	0	18	26
Patients with antibiotic treatment, N (%)	3 (75.0)	4 (100.0)	0	9 (50.0)	16 (61.5)
Rate of treatment episodes per person-year	3.20	2.58	0	1.48	1.89
Rate of treatment days per person-year	29.62	45.20	0	30.01	32.23
Systemic antibiotic use					
Overall, N	4	11	8	38	61
Patients with antibiotic treatment, N (%)	3 (75.0)	7 (63.6)	4 (50.0)	26 (68.4)	40 (65.6)
Rate of treatment episodes per person-year	3.20	1.27	1.41	2.13	1.99
Rate of treatment days per person-year	29.61	48.72	96.01	31.53	39.62
North America, N	0	7	8	20	35
Patients with antibiotic treatment, N (%)	0	3 (42.9)	4 (50.0)	18 (90.0)	25 (71.4)
Rate of treatment episodes per person-year	0	0.95	1.41	2.96	2.29
Rate of treatment days per person-year	0	53.40	96.01	48.82	56.79
Europe, N	4	4	0	18	26
Patients with antibiotic treatment, N (%)	3 (75.0)	4 (100.0)	0	8 (44.4)	15 (57.7)
Rate of treatment episodes per person-year	3.20	1.81	0	1.31	1.66
Rate of treatment days per person-year	29.62	41.07	0	14.27	20.49

N, number of patients.

In the published article, there was an error in **Figure 5** as published. The image shows the number of infusions administered at the rate of 20–30 mL/h with no ISRs to be 238. The correct number is 228. The corrected **Figure 5** and its caption “Infusion flow rate and infusion site reactions. ISR, infusion site reaction” appear below.

**Figure 5:**
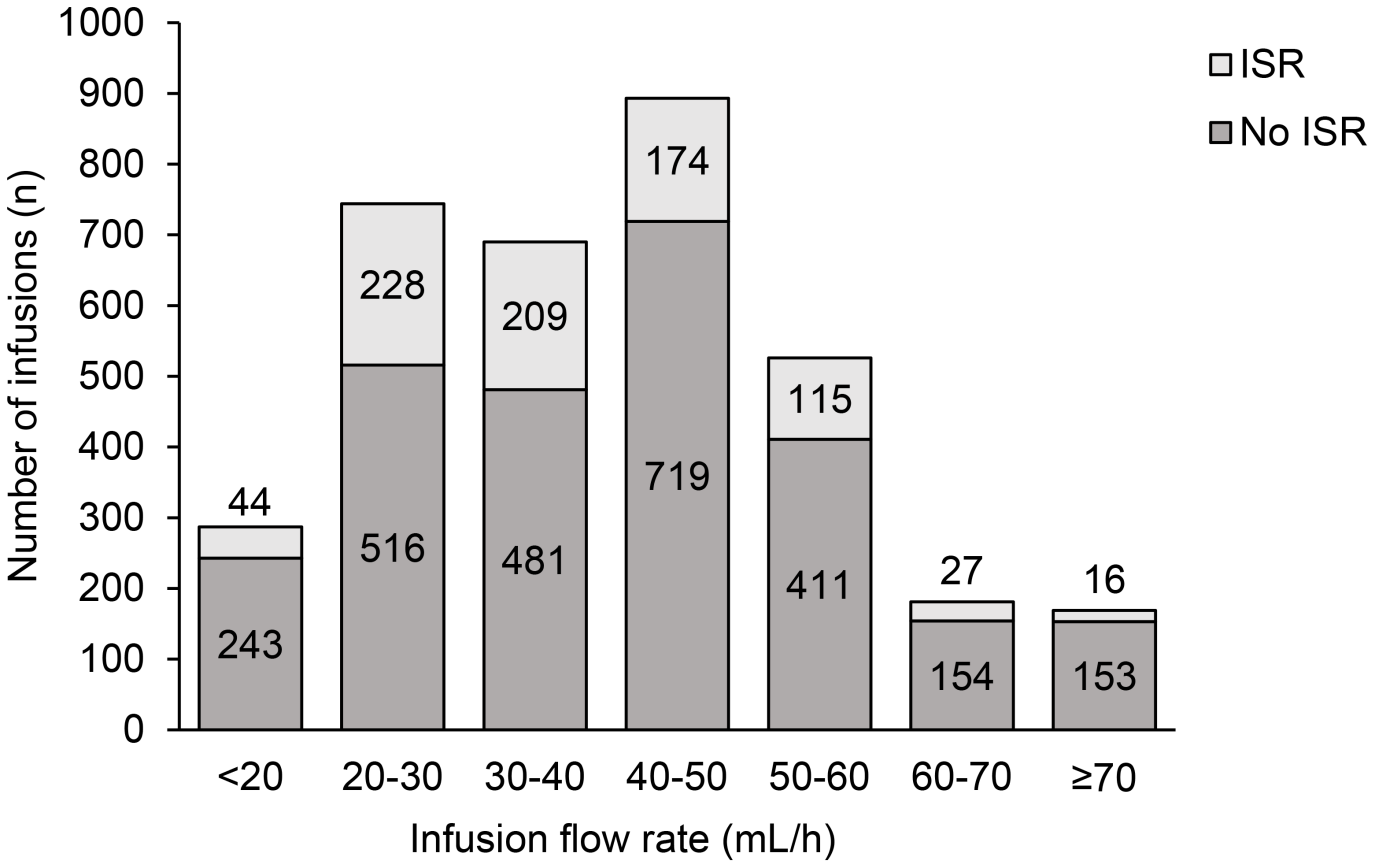
Infusion flow rate and infusion site reactions.

A correction has been made to **the Results section**, *SCIG Administration Characteristics*, Paragraph 1. These sentences previously stated:

“The mean infusion flow rate was 23.86 mL/h/site and was likewise lower in children and adolescents (4.19–16.25 mL/h/site). As a general trend, the dose of octanorm per kg, duration of infusions, infusion volume, and infusion flow rate increased with age (Table 2).”

The corrected sentences appear below:

“The mean infusion flow rate was 22.86 mL/h/site and was likewise lower in children and adolescents (14.19–16.85 mL/h/site). As a general trend, the dose of octanorm per kg, duration of infusions, infusion volume, and infusion flow rate increased with age (Table 2).”

A correction has been made to **the Results section**, *Infusion Site Reactions*, Paragraph 1. This sentences previously stated:

“No localized site reactions were observed for three-quarters (76.7%; 814/3497) of analyzed infusions.”

The corrected sentence appears below:

“No localized site reactions were observed for three-quarters (76.7%; 2683/3497) of analyzed infusions.”

A correction has been made to **the Discussion section**, Paragraph 8. This sentence previously stated:

“In the current study, 0.23% of infusions were associated with an infusion site reaction.”

The corrected sentence appears below:

“In the current study, 23% of infusions were associated with an infusion site reaction.”

A correction has been made to **the Disclosure section**, Paragraph 1. This sentence previously stated:

“RK reports… …grants from Vietnam National Children and Hospital Hanoi, Vietnam…”

The corrected sentence appears below:

“RK reports… …grants from Vietnam National Children’s Hospital Hanoi, Vietnam…”

The authors apologize for these errors and state that this does not change the scientific conclusions of the article in any way. The original article has been updated.

## Publisher’s note

All claims expressed in this article are solely those of the authors and do not necessarily represent those of their affiliated organizations, or those of the publisher, the editors and the reviewers. Any product that may be evaluated in this article, or claim that may be made by its manufacturer, is not guaranteed or endorsed by the publisher.

