# Supplementary material for: Clinical Efficacy, Safety and Tolerability of a New Subcutaneous Immunoglobulin 16.5% (Octanorm [Cutaquig®]) in the Treatment of Patients With Primary Immunodeficiencies
Source: Front Immunol. 2019 Feb 4;10:40. doi: 10.3389/fimmu.2019.00040 (PMC6369354; doi:10.3389/fimmu.2019.00040)
Supplement: Supplementary file 1 [file Table_1.docx]

**Supplementary Table 1.**

|  | **Children**  **≥2-<5 years**  **N (%)** | **Children**  **≥5-<12 years**  **N (%)** | **Adolescents ≥12-<16 years**  **N (%)** | **Adults**  **≥16-≤75 years**  **N (%)** | **Total**  **All Patients**  **N (%)** |  |
| --- | --- | --- | --- | --- | --- | --- |
| Enrolled (Total Set) | 4 | 11 | 8 | 38 | 61 | |
| Ongoing | 2 (50.0) | 4 (36.4) | 2 (25.0) | 0 (0.0) | 8 (13.1) | |
| Early-terminated | 0 (0.0) | 0 (0.0) | 3 (37.5) | 3 (7.9) | 6 (9.8) | |
| **Completed^#^** | **2 (50.0)** | **7 (63.6)** | **3 (37.5)** | **35 (92.1)** | **47 (77.0)** | |
| Inclusion in study populations |  |  |  |  |  | |
| Safety* | 4 | 11 | 8 | 38 | 61 | |
| FAS* | 4 | 11 | 8 | 38 | 61 | |
| PP† | 4 | 11 | 5 | 37 | 57 | |
| PK‡ | 0 | 2 | 1 | 19 | 22 | |

**Patient disposition with respect to study completion and analysis population.**

^#^ All patients who completed the study received 64 infusions of octanorm.

* Safety population includes all patients who received at least one octanorm infusion. The FAS population was defined according to the intention-to-treat principle and included all patients for whom any post‐baseline data were available.

† PP populations excluded 4 patients who discontinued during the wash-in/wash-out period and did not enter the 52-week primary observation period.

‡ PK population includes patients who had PK data on all three PK time points (PK_IV_, PK_SC1_ and PK_SC2_).

FAS, full analysis set; PK, pharmacokinetics; PP. per-protocol.
