# Supplementary material for: Clinical Efficacy, Safety and Tolerability of a New Subcutaneous Immunoglobulin 16.5% (Octanorm [Cutaquig®]) in the Treatment of Patients With Primary Immunodeficiencies
Source: Front Immunol. 2019 Feb 4;10:40. doi: 10.3389/fimmu.2019.00040 (PMC6369354; doi:10.3389/fimmu.2019.00040)
Supplement: Supplementary file 2 [file Table_2.docx]

**Supplementary Table 2.**

**Age group and number of infusions at data cut-off for patients with pharmacokinetic analysis still ongoing.**

| **Patient** | **Age group** | **Number of infusions at data cut-off** |
| --- | --- | --- |
| 1 | Adolescent | 20 |
| 2 | Child | 39 |
| 3 | Young child | 50 |
| 4 | Child | 53 |
| 5 | Child | 58 |
| 6 | Child | 62 |
| 7 | Young child | 62 |
| 8 | Adolescent | 63 |
